# Emerging Nanoparticle-Based Diagnostics and Therapeutics for Cancer: Innovations and Challenges

**DOI:** 10.3390/pharmaceutics17010070

**Published:** 2025-01-07

**Authors:** Rachitha Puttasiddaiah, Nagaraj Basavegowda, Nityashree Kyathegowdanadoddi Lakshmanagowda, Vinay Basavegowda Raghavendra, Niju Sagar, Kandi Sridhar, Praveen Kumar Dikkala, Maharshi Bhaswant, Kwang-Hyun Baek, Minaxi Sharma

**Affiliations:** 1 Teresian College Research Centre, Teresian College, Siddarthanagar, Mysore 570011, India; 2 Department of Biotechnology, Yeungnam University, Gyeongsan 38541, Republic of Korea; nagarajb@yu.ac.kr; 3 School of Psychological Sciences, Christ University, Bangalore 560073, India; 4 Department of Food Technology, Karpagam Academy of Higher Education (Deemed to be University), Coimbatore 641021, India; 5 Department of Food Technology, Koneru Lakshmaiah Education Foundation, Vaddeswaram 522502, India; 6 New Industry Creation Hatchery Center, Tohoku University, Sendai 9808579, Japan; 7 Center for Molecular and Nanomedical Sciences, Sathyabama Institute of Science and Technology, Chennai 600119, India; 8 Research Centre for Life Science and Healthcare, Nottingham Ningbo China Beacons of Excellence Research and Innovation Institute (CBI), University of Nottingham Ningbo China, Ningbo 315000, China

**Keywords:** nano-oncology, nano-omics, graphene, dendrimers, lipid nanoparticles

## Abstract

Malignant growth is expected to surpass other significant causes of death as one of the top reasons for dismalness and mortality worldwide. According to a World Health Organization (WHO) study, this illness causes approximately between 9 and 10 million instances of deaths annually. Chemotherapy, radiation, and surgery are the three main methods of treating cancer. These methods seek to completely eradicate all cancer cells while having the fewest possible unintended impacts on healthy cell types. Owing to the lack of target selectivity, the majority of medications have substantial side effects. On the other hand, nanomaterials have transformed the identification, diagnosis, and management of cancer. Nanostructures with biomimetic properties have been grown as of late, fully intent on observing and treating the sickness. These nanostructures are expected to be consumed by growth in areas with profound disease. Furthermore, because of their extraordinary physicochemical properties, which incorporate nanoscale aspects, a more prominent surface region, explicit geometrical features, and the ability to embody different substances within or on their outside surfaces, nanostructures are remarkable nano-vehicles for conveying restorative specialists to their designated regions. This review discusses recent developments in nanostructured materials such as graphene, dendrimers, cell-penetrating peptide nanoparticles, nanoliposomes, lipid nanoparticles, magnetic nanoparticles, and nano-omics in the diagnosis and management of cancer.

## 1. Introduction

The field of cancer diagnosis and management has experienced transformative progress with the development of multifunctional nanoparticles. These cutting-edge technologies offer combined diagnostic, therapeutic, and monitoring capabilities, enabling precise oncology and reducing systemic toxicity. Multifunctional nanoparticles, such as graphene-based nanomaterials (GBNMs), gold nanoparticles, and magnetic nanoparticles, have been widely studied for their high biocompatibility, large surface area-to-volume ratios, and versatile functionalities [1].

Recent advancements include dual-targeting nanomedicine integrating photothermal and chemotherapy, which demonstrates enhanced delivery to tumor cells with minimal impact on healthy tissues. Nanocarrier-encapsulating drugs and diagnostic probes have emerged as promising platforms for the treatment of diseases such as breast, prostate, and liver cancers. Additionally, metal and metal oxide nanoparticles have shown significant potential for overcoming drug resistance and achieving higher therapeutic efficacy [2].

The development of biosensors based on multifunctional nanoparticles has revolutionized early cancer detection. These sensors demonstrate enhanced sensitivity and specificity for biomarkers, such as alpha-fetoprotein (AFP) and the vascular endothelial growth factor (VEGF), ensuring accurate diagnosis at earlier stages. Moreover, nanotechnology has facilitated real-time imaging and targeted drug delivery through techniques such as theranostics, thereby addressing critical gaps in cancer treatment [3].

Cancer cells protect against cell death (apoptosis), uncontrolled growth, cellular signaling, invasion, metastasis, and angiogenesis. Cancer typically starts as a localized tumor that has the potential to spread (metastasize) to other parts of the body, making its management challenging. Cancer mortality and morbidity rates are increasing worldwide. Global cancer severity, frailty, and prevalence (GLOBOCAN) 2018 data showed that more than 18.1 million new cases of cancer were anticipated, along with 9.6 million cancer-related deaths. In 2020, the World Health Organization (WHO) predicted that there would be over 19.3 million new cancer cases and 10 million cancer-related deaths. After 2030, it is anticipated that 30 million people will perish from cancer annually [4]. Over the years, various cancers have evolved, along with their corresponding treatments and associated survival rates (Table 1).

**Figure 1:**
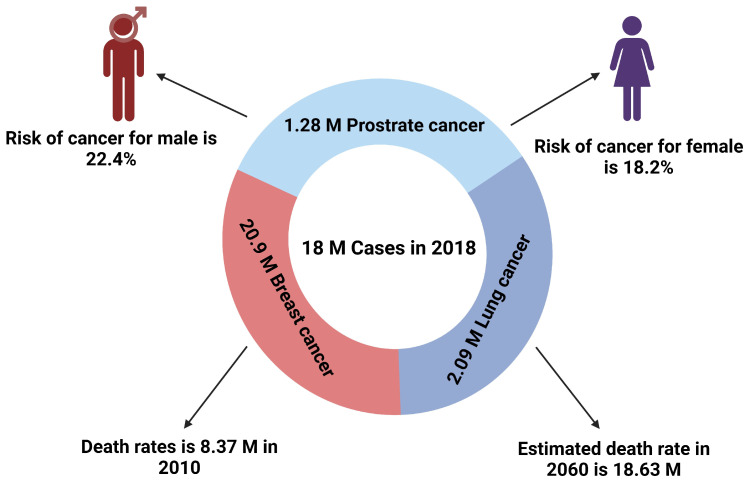
The statistics of the risk of cancer in both males and females according to the World Health Organization (WHO). This figure is redrawn from Mattiuzzi and Lippi [5] and is an open access article (copyright © 2019 Atlantis Press International B.V., Dordrecht, the Netherlands) distributed under the terms and conditions of the Creative Commons Attribution (CC BY)-No Commercial 4 license.

The American Cancer Society and the World Health Organization give recent data on the survival, death, and prevalence rates of the 15 most prevalent malignancies globally. Malignant growth is the human infection with the highest clinical, social, and economical costs in terms of cause-explicit ability altered life years (DALYs). According to Figure 1, the risk of malignant development was 20.2% for those aged 0–74 (22.4% for males and 18.2% for women, respectively). The majority of the 18 million new cases of malignant development that were examined in 2018 were in the lung, breast, and prostate (2.09 million, 20.9 million, and 1.28 million, respectively). With the exception of sex-explicit cancers (i.e., 0.30), the recurrence proportion across individuals is more significant than one for all cancers, save thyroid cancer. After ischemic coronary disorders, malignant growth is presently the world’s largest cause of mortality (8.97 million deaths). But, by 2060, malignant growth is expected to overtake it (18.63 million passings). Lung, liver, and stomach cancers are the three most lethal malignancies in humans, and lung and breast cancers are the leading causes of mortality. Prostate and thyroid cancers have a 100% 5-year endurance rate, but esophageal, liver, and pancreatic cancers typically have a 20% 5-year endurance rate [5,6,7].

**Table 1:** Types of cancer, the use of anti-tumor drugs/surgery, and the survival rate of today compared to 5, 10, and 20 years ago [8,9,10,11,12].

Cancer Type	Common Treatments	5-Year Survival Rate (Today)	5-Year Survival Rate (20 Years Ago)
Breast cancer	Surgery, Chemotherapy, Radiation, Hormone Therapy, Immunotherapy	91%	77%
Prostate cancer	Surgery, Radiation, Hormone Therapy, Chemotherapy	98%	87%
Lung cancer	Surgery, Chemotherapy, Radiation, Immunotherapy, Targeted Therapy	25%	16%
Colon cancer	Surgery, Chemotherapy, Radiation, Immunotherapy	65%	58%

The recent statistics in India (2023–2024) clearly show the cancer incidence in 2022, of which approximately 1.46 million new cancer cases were recorded in India, with an estimated increase of 12.8% by 2025. This corresponds to a crude incidence rate of 100.4 cases per 100,000 individuals. Approximately one in nine Indians is likely to face a cancer diagnosis during their lifetime. Breast cancer is the most common cancer among Indian women, accounting for 25–32% of all female cancers, especially in urban areas such as Delhi, Mumbai, and Bengaluru. Among men, lung cancer ranks highest, largely attributed to tobacco use. Regional variations, such as Kerala, have the highest cancer incidence rate (135.3 cases per 100,000), followed by Mizoram (121.7) and Haryana (103.4). States such as Uttar Pradesh, Maharashtra, and West Bengal have reported the highest absolute number of cancer cases. Childhood cancers (ages 0–14 years) comprise 4% of all total cases, with lymphoid leukemia being the most common in boys and girls. The lack of specialized pediatric oncology program is a significant challenge in addressing this issue. Cancer accounts for 18.1% of deaths among non-communicable diseases in India in 2022. The burden of cancer, measured in disability-adjusted life years (DALYs), is projected to increase from 26.7 million in 2021 to 29.8 million by 2025. Major challenges and efforts are limited healthcare access in rural areas, leading to late-stage diagnoses, particularly in lung, gallbladder, and prostate cancers. Public health initiatives such as the Ayushman Bharat-Pradhan Mantri Jan Arogya Yojana (AB-PMJAY) aim to make cancer care more accessible and affordable [13].

Therefore, the ongoing monster-like state of cancer has forced scientists to discover various strategies for accurate cancer diagnosis and therapy, and nano-oncology represents an emerging therapeutic modality with the potential to treat cancer [14]. Nanotechnology is a branch of nanomedicine that uses nanotechnology for cancer administration and treatment. The use of nanotechnology in biomedical science has expanded owing to advancements in the field [15]. Healthcare strategies are being altered by nanotechnology, which is anticipated to have a significant impact on the years to come and improve healthcare facilities. This has contributed to both diagnostics and the viability of therapeutic drug delivery. Clinical nanotechnology and nano-pharmacology concentrate on the planning, creation, control, and utilization of helpful medications and gadgets made of materials at the nanoscale (1–100 nm) [16]. The capability of nanoparticle-based drug conveyance has been applied to gene therapy and the conveyance of additional perplexing particles to the exact site of activity. Malignant growth, diabetes, infectious diseases, neurodegenerative diseases, blood issues, and orthopedic diseases are among the circumstances that can benefit from nano-based drug delivery frameworks. In addition, the development of multifunctional nanotherapeutics could possibly close these holes in the ongoing remedial field.

Since nanomedicines can efficiently deliver the treatment to the affected tissues, lowering drug damage, they are more successful than existing restorative techniques at curing malignant growth. Therefore, polymeric paclitaxel micelles and polymeric asparaginase forms have been suggested recently for the treatment of a number of cancerous diseases. Nanotechnology-based medicines and diagnostics are more effective and pose little or no toxicity risks. Likewise, there is potential for future uses of more recent nano-level imaging components for diagnostic imaging. Nanoparticles are now being used for various healthcare applications, thanks to advancements in nanotechnology. Precision diagnostics, less invasive surgery, and targeted medication administration are all made possible by nanorobotics, a State-of-the-Art development in nanotechnology that is revolutionizing healthcare. Especially in the treatment of cancer, these tiny devices can transport therapeutic compounds directly to sick tissues, reducing side effects and increasing efficacy [17]. In order to treat neurological conditions like Parkinson’s and Alzheimer’s, they are also being developed for microsurgeries that include removing blockages in the arteries or bridging the blood–brain barrier. Furthermore, by using sophisticated imaging and biomarker interactions, nanorobots are showing promise in the fight against antibiotic-resistant infections and in the early detection of cancer cells [18]. Nanorobotics is anticipated to transform personalized medicine and enhance patient outcomes by incorporating artificial intelligence and accelerating clinical trials.

Nano-vehicles are nanomaterials that can serve as delivery systems for conjugated drugs. A drug delivery system based on nano-vehicles used in cancer therapy specifically targets tumor cells and the microenvironment that supports cancer cells. Nano-vehicles with therapeutic drugs payloads for the selective targeting and killing of tumor cells, the selective delivery of drugs to tumor cells, cancer stem/tumor-initiating cells, and/or a favorable microenvironment for cancer cell growth [19,20]. This review summarizes the evolution of nanostructured materials for cancer treatment. Academic publications, including books, book chapters, reviews, research articles, and conference proceedings, were sourced from the online platforms Web of Science™ (updated as of 25 December 2024) and Google™ Scholar. A comprehensive search was performed using search strings, such as cancer epidemiology, nanotechnology in cancer therapy, nano-oncology, nanostructures, dendrimers, graphene, cell-penetrating peptide nanoparticles, antibody-conjugated nanoparticles, lipid nanocarriers, magnetic nanomaterials, nano-omics, and environmental factors and outcomes. All publications retrieved were restricted to those written and published in English, totaling 204 documents.

## 2. Nanotechnology Approach in Cancer Therapy

Nano-oncology is a new interdisciplinary research area that spans biology, chemistry, engineering, and medicine. This is expected to significantly improve cancer diagnosis, treatment, and prevention [21]. The investigation of nanotechnology extends to the disciplines of biology, chemistry, physics, engineering, and medicine. One likely use for nanotechnology in oncological sicknesses is the improvement of multifunctional nanoparticles that can target disease cells, convey and deliver drugs in a controlled manner, and distinguish malignant growth cells very explicitly and delicately [22]. Many medicines that are both time and cash consuming are accessible today. Nanotechnology can be used to develop quicker and more affordable medicines. The application of nanotechnology to medication has another viewpoint. The medication can be unequivocally designated by utilizing nanotechnology, expanding its viability, and reducing the probability of any likely incidental effects [23]. Although various medications are available to treat cancer, utilizing a nanotech-based approach increases action while reducing incidental effects [24]. Malignant growth is the most widely recognized infection. We refer to the nanotech-based approach in this audit, particularly the utilization of nanoparticles (NPs) and their different structures in conveying anticancer medications [25]. Chemotherapeutic drugs can be delivered directly to tumors using NPs, sparing healthy tissues [26]. Compared with conventional chemotherapy, nanocarriers offer several advantages.

**Site-specific targeting:** Nanoparticle-based drug delivery systems have unique physical, chemical, and biological properties that enhance the site-specific delivery of chemotherapeutic agents [27]. Recent studies have also emphasized the role of functionalized nanoparticles in improving localization to tumor sites while minimizing off-target effects [28].

**EPR effect:** Nanoparticles can accumulate preferentially in tumor tissues owing to their size and surface characteristics through the enhanced permeability and retention (EPR) effect. Updated research highlights advancements in exploiting the EPR effect through NP surface modification, enhancing cancer-targeting efficiency [29].

**Therapeutic versatility:** NPs can deliver a variety of anticancer agents, including small molecules, peptides, proteins, and genes. They also modulate redox responses, systemic distribution, and drug release kinetics. Recent breakthroughs include stimuli-responsive NPs that release drugs under specific conditions such as pH, temperature, or enzyme presence [30].

**Biodegradable polymeric NPs:** Solid particles of biocompatible polymers (< 1000 nm). Newer developments have focused on hybrid nanocarriers that combine these materials to optimize drug delivery [31].

**PLGA nanocarriers:** Poly (lactic-co-glycolic acid) (PLGA) nanocarriers improve issues such as short circulation half-life and non-specific targeting. Recent findings suggest that these systems can enable the co-delivery of multiple drugs or therapeutic agents, thereby enhancing their efficacy against resistant cancer types [32].

**Folate receptor targeting:** Cancer cells in tissues such as the brain, kidney, breast, ovary, and lung often overexpress folate receptors. Nanoparticles functionalized with folic acid can selectively target these cells. Advances in this approach have shown promise in clinical trials by increasing the precision of targeting [33].

Tumor targeting is one of the profound benefits of nanotechnology in cancer treatment. The goal of nanotechnology in cancer treatment is to distinguish between malignant cells and selectively eliminate them. Passive and active targeting are the key mechanisms that distinguish malignant from non-malignant cells. To increase the concentration of NPs in tumors, passive targeting uses the enhanced permeability and retention (EPR) effect [34]. For the treatment and management of numerous diseases, RNA-based therapeutics such as messenger RNA (mRNA), microRNA, and small interfering RNA (siRNA) have become increasingly popular over the past 20 years (e.g., cancers, diabetes, inflammatory diseases, genetic disorders, and neurodegenerative diseases) [35]. The nanoparticles move throughout the body, look for epigenetic changes linked to cancer, help with imaging, release a therapeutic agent, and assess how well the intervention works [36]. By identifying certain biomarkers on their surface, nanorobots can precisely administer medications to cancer cells while sparing healthy tissues [37]. Once attached to cancer cells, nanorobots can produce heat or release cytotoxic chemicals to cause necrosis or apoptosis, which kills the cells. By changing DNA methylation and histone modification patterns, nanorobots can transport DNA or RNA molecules that mute oncogenes or reactivate tumor-suppressing genes [38]. To ensure targeted therapy and reduce side effects, these payloads are delivered in response to stimuli such as pH changes or enzymes in the tumor environment [39]. Nanorobotics is a potent tool for gene control and targeted cancer therapy because of its accuracy.

**Figure 2:**
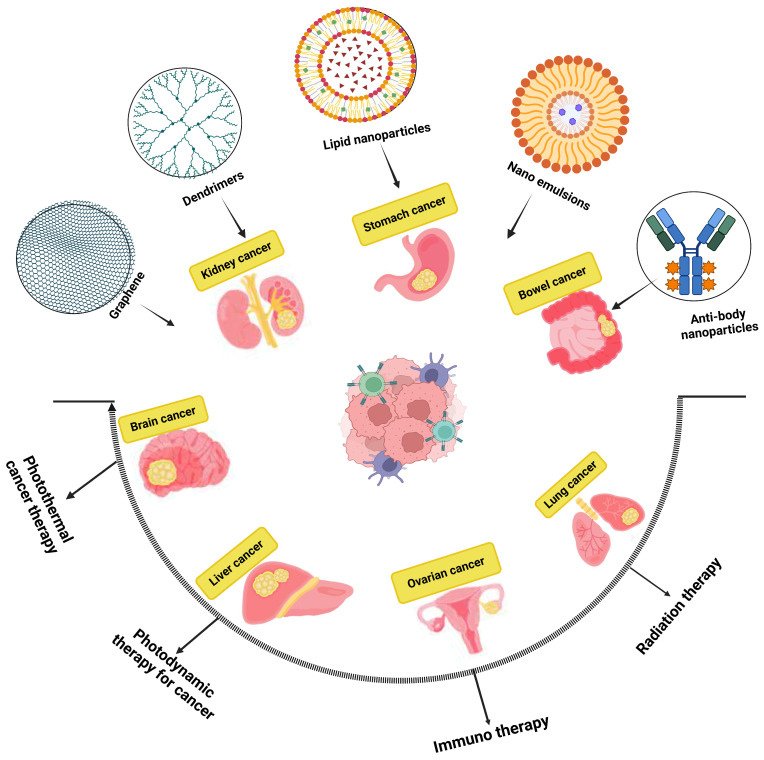
The different nanoparticles for the treatment of cancer. This figure is constructed by the authors using BioRender, Toronto, Ontario, Canada.

Various methodologies of nanomedicine using dendrimers, quantum dots, and polymeric/non-polymeric NPs have been developed, and numerous studies have been conducted on nanoparticles made of lipids, carbon nanotubes, and micelles. Additionally, multivalent ligand targeting and the ability of nanomedicines to transmit a large payload are crucial in cancer treatment. This enables the targeting of specific tissues while avoiding defense mechanisms. The potential toxicity of these pharmaceuticals is a major challenge. Before nanomedicines can be used as cancer treatments, owing to the presence of NPs, a thorough and extensive assessment is essential [40]. The development of a biocompatible nanosystem is essential for conveying nanomaterial-formed medications to the designated growth site. Such nanosystems include areas of strength for nanocrystal NPs, nanostructured lipid transporters, lipid drug forms, nanoliposomes, dendrimers, nanoshells, emulsions, nanotubes, and quantum spots (Figure 2).

Uninvolved and dynamic methodologies have been utilized in the conveyance of nanomaterials and drug edifices, and they can also be applied to the conveyance of nanodrugs. Latent focusing is dependent on the vasculature encompassing the improved porousness and upkeep (EPR) impacts of cancers. Drug transportation through dynamic focusing involves the ligand-composed restriction of NPs to receptors tracked down on cancer cells. Drug discharge from NPs that has been stacked with medications may be controlled by physiological reactions such as temperature and pH. Variable bioavailability profiles and the chemotherapeutic viability of nano-drugs in vivo are brought about by an assortment of nanomaterial properties, including their size, surface charge, PEGylation, and other biophysical properties [41].

The emergence of nanomedicine as a cutting-edge and potential substitute technology has several advantages over conventional cancer treatments and opens up new avenues for precise cancer diagnostics, improved cancer therapy, and early detection. Cancer complexity and dynamics must be taken into account in order to bridge the translational bench-to-bedside gap, even if cancer nanomedicines can deliver chemotherapeutic drugs with low systemic toxicity. To benefit from the tumor microenvironment and learn more about the fundamental biological processes that underlie cancer and how they impact blood flow, tumor penetration, and interactions between proteins and nanoparticles, it is imperative to do appropriate research [42].

### 2.1. Dendrimers

**Figure 3:**
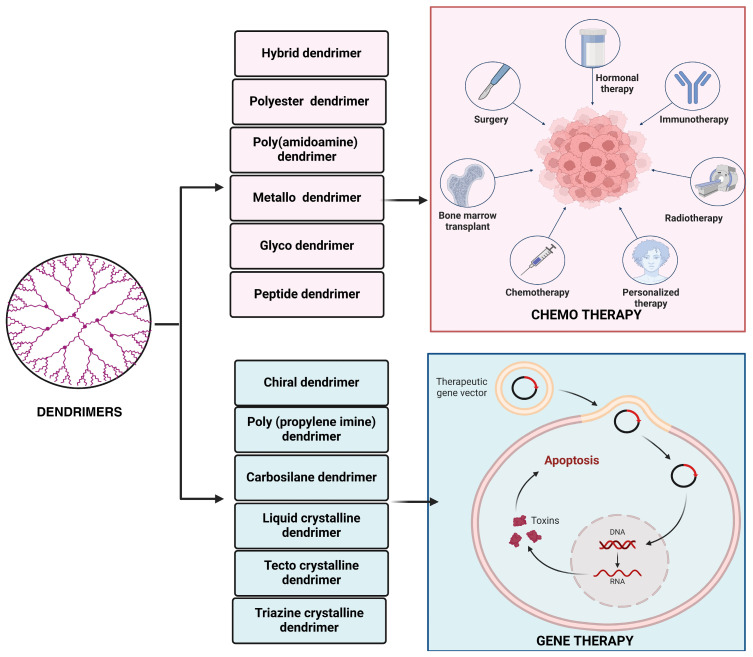
Dendrimers and their different types for chemotherapy and gene therapy. This figure is constructed by the authors using BioRender, Toronto, Ontario, Canada.

Dendrimers have a globular shape and can easily polymerize their surfaces in a controlled manner. These qualities make these structures excellent candidates for use in drug delivery systems [43]. Based on their functionalized moieties, dendrimers are divided into different categories, including PAMAM [Poly (amidoamine) dendrimer], PPI [poly (propylene imine) dendrimer], liquid crystalline, chiral, core–shell, peptide, glycodendrimers, with the PAMAM as the most researched for oral drug delivery because of their water solubility and ability to pass through epithelial tissue, enhancing their transfer via the paracellular pathway (Figure 3) [44].

Simple encapsulation, covalent conjugation, and electrostatic interactions are the three main mechanisms used to load drugs into dendrimers [45]. The biocompatibility of dendrimers has been used to deliver potent drugs, such as doxorubicin. This nanostructure attaches ligands to the surfaces of cancerous cells to target them (Table 2) [46].

### 2.2. Graphene

Graphene-based nanomaterials (GBNMs) have been contemplated and utilized in numerous biomedical fields, especially in disease therapy. These are increasingly being considered because of their upgraded restorative adequacy and reduced antagonistic impacts. GBNMs, which are conventional two-layered (2D) nanomaterials, have an unmistakable construction and remarkable physicochemical characteristics, making them very encouraging for disease therapy. One molecule of sp2-bound carbon particles is stuffed together to shape a planar sheet, which is the premise of graphene [67]. Graphene oxide is hydrophilic because of the practical social affairs containing oxygen that are open on the edge and basal planes [68]. This property makes graphene oxide dispersible in water and different regular solvents [69].

**Figure 4:**
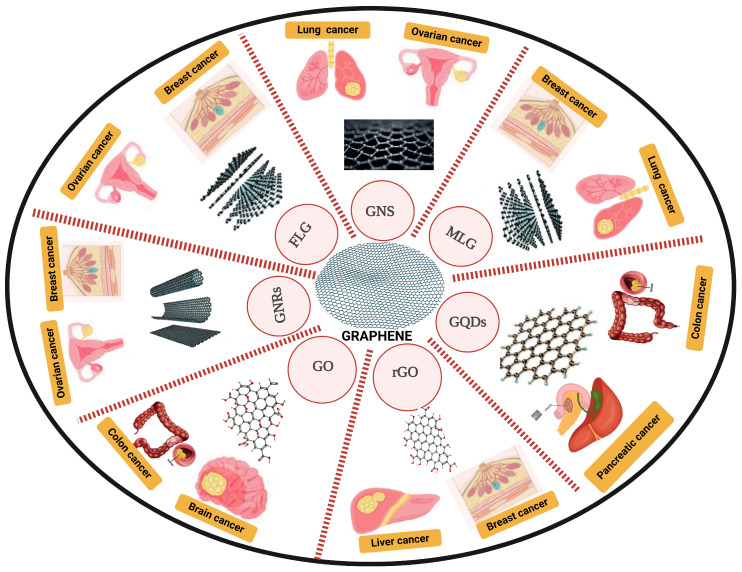
Graphene-based nanomaterials in the treatment of cancer. GNRs, graphene nanoribbons; GNSs, graphene nanosheets; FLG, few-layer graphene; MLG, multilayer graphene nanoplatelets; GO, graphene oxide; rGO, reduced graphene oxide; GQDs, graphene quantum dots. This figure is constructed by the authors using BioRender, Toronto, Ontario, Canada.

The most moderate particles utilized for the covalent distinction in graphene’s surface are polyethylene glycol (Stake), folic destructive, chitosan, poly (vinyl liquor) PVA, and polyethyleneimine (PEI), which can improve graphene and graphene oxide’s biocompatibility in physiological medium [70]. GBNMs are extraordinarily flexible and make great nanoplatforms for nanobiosensors because of their fantastic charge portability, high surface region to volume proportion, mathematical design, and optical and electrical properties (nanoscale biosensors). Therefore, they play a critical role in numerous aspects of malignant growth. To improve early cancer diagnosis and screening, graphene-based nano-biosensors increase the limit of detection, are highly sensitive and specific, and are capable of simultaneously detecting multiple cancer biomarkers (Figure 4) [71].

Alpha-fetoprotein (AFP) is a malignant growth marker for hepatocellular carcinoma and an oncofetal glycoprotein that is encoded by the AFP quality [72]. An imprint-free amperometric immunosensor for AFP was left using a composite film with a smooth carbon terminal with TiO_2_, graphene, chitosan, and gold nanomaterials. High selectivity, responsiveness, and precision were observed over a wide area (0.1300 ng/mL). In addition, nitrogen-doped carbon quantum spots (luminophores) and aminated graphene (extinguishing normal for discretionary neutralizers), a sandwich-type electrochemiluminescence immunosensor, were developed [73]. The immunosensor had a discovery cut-off of 3.3 pg/mL and an identification scope of 0.01–100 ng/mL [74]. A quantitative electrochemical immunosensor (Cu_2_O@GO) for AFP recognition consolidates cuprous oxide nanowires with graphene oxide nanosheets. They focused on the Cu_2_O@GO nanocomposite as a decent contender for immunosensors and found that electrochemical immunosensors were profoundly specific and explicit [75].

One growth marker for ovarian illness is sugar antigen 125 (MUC16), a glycoprotein that is encoded by MUC16. A sandwich-style electrochemiluminescence immunosensor was fabricated to track down low levels of carb antigen 125 in biological arrangements. As a nanoplatform, they utilized center shell Fe_3_O_4_-Au nanomaterials, and, to support the sign, they functionalized graphene with quantum specks. They emphasized that this device provides a broad identification range (0.00550 U/mL) and a location cut-off of 1.2 mU/mL for carb antigen 125 [76].

The transmembrane glycoprotein encoded by sugar antigen 153 (MUC1) is an electrochemical immunosensor that is the prognostic biomarker for dangerous chest development. The assistant carb antigen 153 checking specialist is attached to cadmium-functionalized nanoporous TiO_2_, while the basic sugar antigen 153 neutralizers are associated with functionalized graphene. They spread out an extensive ID window for sugar antigen 153 (0.0260 U/mL) and a low area breaking point of 0.008 U/mL [77]. A tumor marker for a number of solid malignancies, such as colorectal, breast, and pancreatic tumors, is the carcinoembryonic antigen. Cell connections are made by this glycoprotein. To detect carcinoembryonic antigens, an ultrasensitive luminol electrochemiluminescence immunosensor of the sandwich type was first developed. It used ZnO nanomaterials for aesthetics and graphene as a platform. It was linked to glucose oxidase and an optional vaccine. With a recuperation rate ranging from 89% to 108%, this immunosensor exhibited high exactness. Its precision was equivalent to that of the ELISA units, which had a general blunder pace of less than 5%. Protein frequently overexpressed in cancers, particularly in kidney cancer (RCC). MUC1’s abnormal behavior in cancer cells can drive tumor growth, promote cell invasion, and contribute to resistance to treatment [78]. Nanomaterials offer unique properties that enhance biosensor performance, including increased sensitivity, stability, and selectivity. These materials are crucial for developing point-of-care devices that can accurately detect low-concentration cancer biomarkers in biological fluids, enabling precise and timely cancer diagnosis [79]. highly sensitive and label-free carbon nanotubes-based field-effect transistor biosensor for rapid endotoxin detection. By incorporating carboxylated graphene quantum dots and polymyxin B, the sensor achieved exceptional sensitivity with low detection limits in both PBS and serum, enabling early diagnosis of Gram-negative bacterial infections in blood samples with high accuracy [80]. This review focuses on non-enzymatic electrochemical glucose sensors, exploring the electrocatalytic oxidation mechanism and evaluating the performance of various electrode materials, including noble metals, transition metals, and bimetallic oxides. The review highlights the advantages of non-enzymatic sensors, such as enhanced stability compared to enzyme-based sensors, while also discussing the challenges and future directions for this promising technology in glucose sensing applications [81]. A nanomaterial mix of graphene, attractive globules, and gold can be recognized (Table 3).

For epithelial carcinomas, the squamous cell carcinoma antigen serves as a cancer marker. A extremely delicate sandwich-type electrochemical immunological sensor was used to detect the expression of both the carcinoembryonic antigen and the squamous cell carcinoma antigen in cervical carcinomas. They employed the CMK-3 neutralizer redox to designate the gold@mesoporous carbon and utilized decreased graphene oxide-tetramethylene pentamine as a substrate. With recovery rates of 96% and 104%, this nanobiosensor was outstandingly careful and delicate, with high responsiveness [99]. The prostate-explicit antigen is a biomarker for prostate sicknesses and a glycoprotein catalyst encoded by the KLK3. While diagnosing malignant growth of the prostate and observing treatment, it is particularly useful. A graphene-based gold nanocomposite immune sensor with a three-layered electrochemical construction was used for the mark-free recognition of prostate-explicit antigens. With a discovery cut-off of 0.59 ng/mL and a recognition scope of 0–10 ng/mL, this immune sensor showed high selectivity and strength [100]. The creation of an electrochemical immunity sensor involved combining a silver-hybridized mesoporous silica nanomaterial with an amino-functionalized graphene sheet ferrocene carboxaldehyde nanocomposite. In contrast to the immune sensor and ELISA unit, the general standard deviation increased from 4.61% to 4.10%. Furthermore, it has a recognition breaking point of 2 pg/mL [101].

Vascular endothelial development factors direct angiogenesis and vasculogenesis. It is regularly overexpressed, particularly in the hypoxic microenvironments of strong cancers that progress at this stage. A recombinant refined monoclonal neutralizer and a designated medication specifically designed to inhibit vascular endothelial development factor-A, bevacizumab (Avastin), was employed as a biorecognition component in an electrochemical biosensor that was primarily based on an appealing graphene oxide-modified gold terminal. The Avastin-connected attractive graphene oxide electrochemical biosensor, which had an identification scope of 31.25 to 2000 pg/mL and beat ELISA units with regard to reusability, cost viability, and high selectivity for the location of vascular endothelial development factor, was underlined by the creators [102].

### 2.3. Cell-Penetrating Peptide (CPP) Nanoparticles

Nanoscale material treatments have been developed to address current pharmacological limitations. The area of nanoparticle pharmacokinetics is the primary emphasis to obtain optimal performance with efficient absorption and an appropriate pharmacokinetic profile [103]. Nanoparticle optimization with surface changes is required to provide far more effective and productive therapy. Several modifications have been investigated for drug targeting, which is either passive or active. For example, extended circulation times and reduced phagocytosis have been associated with coating nanoparticles with hydrophobic substances such as PEG, poloxamers, and polysorbate 80 (Tween 80). Furthermore, peptides (CPP-avidin-biotin), immunoglobulins, transferrins, and saccharides (mannose, hyaluronic acid), among other substances, can be used in the synthesis and characterization of nanoparticles to produce specific interactions between the target ligand and nanoparticle conjugates [104].

**Figure 5:**
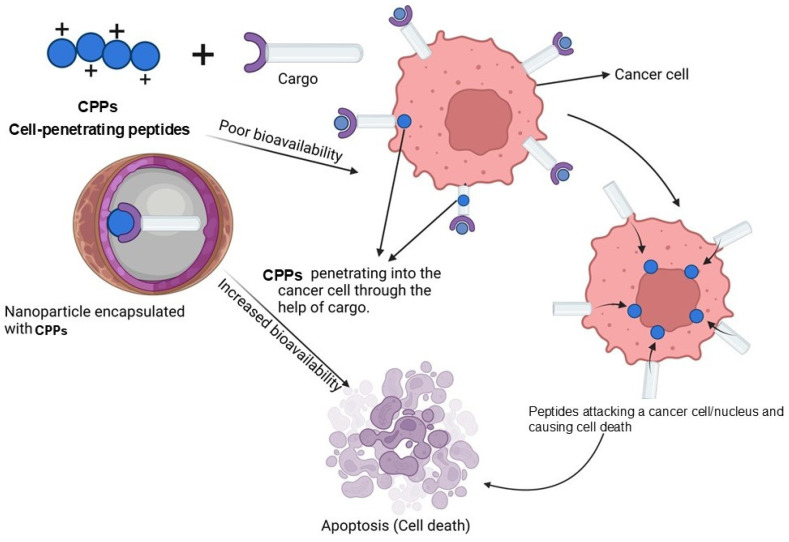
CPP nanoparticles induced apoptosis mechanism. This figure is constructed by the authors using BioRender, Toronto, Ontario, Canada.

CPP nanoparticles play a role in enhancing bioavailability by employing lipids. The intracellular distribution of pharmaceutical substances is one of the main challenges in effective therapy. Proteins and other substances are inhibited by the biological cell membrane unless an active transport mechanism is present. Predefined pharmaceutical nanocarriers have been created to hasten the transport of drugs inside cells and increase the stability of such drugs once they have been administered (Figure 5). Although CPPs and nanoparticles are transportation vectors with the greatest promise, there are still certain limitations. The conjugation of CPPs with NPs has been investigated as a way to bridge the gap between the two molecules and to accelerate the development of new chemicals or conjugates with better effectiveness, precision, and therapeutic activity (Table 4). Several studies have demonstrated the efficacy of using this conjugation topically to treat a various ailments, including dermatological disorders.

Amphipathic CPPs often have a nonpolar area that is rich in hydrophobic amino acids (such as valine, alanine, leucine, and isoleucine) and a cationic, anionic, or polar region. The appropriation of these areas with different polarities fluctuates in the essential and optional designs of amphipathic peptides [120,121]. The sequence or the folding of CPP into an α-helix with a hydrophilic and hydrophobic face led to amphipathicity [122]. The presence of many hydrophobic residues in the structure may be one of the main contributors to the low net charge and high cell-membrane binding of hydrophobic CPPs [123]. If not, CPP would become insoluble in physiological liquids and would get caught in the hydrophobic center of the lipid bilayer after passing through the cell layer. Hydrophilic amino acids were also added to the mixture. An alternate degree of categorization is essential from the clinical perspective. CPPs are isolated into peptides that are special to cells and those that are not. Cell-explicit CPPs, as their names plainly infer, can convey freights to specific cells, yet non-cell-explicit CPPs cannot [124].

### 2.4. Antibody Conjugated Nanoparticles

The success of nanotechnology and antibody therapies serves as the foundation for antibody-conjugated NP (ACNP) strategies. Controlled drug delivery, preservation of the drug’s chemical structure, the decreased risk of secondary metabolites (if metabolism is unpredictable), and possibly lower toxicity are some of the advantages of developing ACNPs over developing ADCs [125]. Antibody-conjugated nanomaterials can be used for photothermal therapy (PTT), targeted drug and gene delivery, and combination therapies. In vivo and in vitro applications fall into the category of diagnostic application. Therapeutic and diagnostic applications are combined in theragnostic systems [126].

Antibodies are the most popular ligands for targeting tumor cells [127]. With respect, ligand-put centering is more uplifting than other idle and actual zeroing in on procedures for quality movement. Ligands associated with the external layer of nanoparticles unequivocally team up with overexpressed cell surface receptors, including folate receptor alpha (FRA), the epidermal improvement factor receptor (EGFR), integrins, CD44, and transferrins, which are known to be overexpressed in diseased cells [128]. ACNPs have several advantages in drug delivery systems because they preserve the chemical structure of drugs, deliver them systematically, and even reduce toxicity [129].

Based on these properties, antibodies that may subsequently be employed as nanoparticle vectors can be created using membrane proteins that are overexpressed in tumor cells. The mechanisms of action of ADCs and ACNPs, two types of targeted treatments, are comparable. Following their attachment to the target endosomes, these complexes are absorbed into the cell by receptor-mediated endocytosis [130].

Later, the drug is released into the cytoplasm by the coupling of endosomes and lysosomes [131]. The polymeric nanoparticles containing the monoclonal antibody trastuzumab, which interacts with the surface protein HER2 present on the surface of all breast cancer cells, start to concentrate in sites where HER2 is overexpressed in breast cancer cells. These particles may also contain taxanes, which work in conjunction with trastuzumab to treat breast tumors. NPs are internalized via receptor-mediated endocytosis [132].

For instance, RGD-adjusted paclitaxel (PTX) and cisplatin (CDDP)-stacked LPNs (RGD-ss-PTX/CDDP LPNs) have zeta possibilities of −35 mV and diameters of around 190 nm in the therapy of cellular breakdown in the lungs. The antitumor movement of LPNs is fundamentally greater than that of free medications, as evidenced by the half-maximal inhibitory fixation (IC50) values of 26.7 and 75.3 μg/mL for pharmaceuticals, stacked LPNs, and free medicine mix [133]. This strategy will help with lung cancer therapy.

To produce antibody-conjugated nanoparticles, sortilin (SORT-1), a human IgG1 monoclonal antibody that selectively encodes caov-4 ovarian malignant cells, was functionalized onto the surface of the particles. Ca(OH)_2_ and TXT were delivered to cancer cells (caov-4 cells) selectively using a novel therapeutic composite consisting of iron oxide nanoparticles (Fe_3_O_4_ NPs) as a biodegradable magnetic core and polyvinyl alcohol (PVA) as an appropriate matrix for drug encapsulation [118]. TiO_2_ NPs were used to create antibody-nanoparticle conjugates (ANCs), which were then characterized using transmission electron microscopy and X-ray photoelectron spectroscopy. Using ultrasound, it was possible to evaluate cell viability, reactive oxygen production, and apoptosis. Reactive oxygen species (ROS) are produced with SDT, cell viabilities are decreased in the presence of ANCs, and glioblastoma cells can be killed using ANC [134]. Preclinical studies across various fields have shown that Ab-targeted NPs can be used to deliver cytotoxic chemotherapy directly to cancer cells, resulting in an effective cure with fewer side effects, and free drugs are contrasted [135].

### 2.5. Lipid Nanoparticles

Lipid materials have been used to develop promising drug delivery nanocarriers. Lipid excipients have a superior biodegradation profile and are mostly made from dietary oils or fats. It can pass through the gastrointestinal barrier in addition to this use. Lipid-based nanocarriers can improve the bioavailability of several medications by reducing hepatic first-pass metabolism, improving lymphatic transport, and reducing P-glycoprotein-mediated efflux [136]. Fatty acids, fatty acid esters, fatty alcohols, natural oils and fats, semisynthetic mono-, di-, and triglycerides, polyethylene glycol derivatives, cholesterol, and phospholipids are among the various types of lipid materials that can be grouped according to their possible application in pharmaceutical relevance [137]. The creation of diverse lipid-based nanocarrier types with a range of applications is made possible by the ability to alter pharmacokinetic properties using lipids. However, a variety of parameters, such as purity, chemical stability, physical and chemical characteristics, solvent capacity, fat digestion, and regulatory requirements, influence the selection of the optimal lipid additive for a certain nanocarrier [138].

Lipid-based nanocarriers are considered a viable medicine delivery method [139]. Lipid nanocarriers can enter cells through membrane fusion without impairing the inherent function of the carrier because they mimic the organic structure of cell membranes and vesicles in nature [140]. The lipid matrix and aqueous phase constitute lipid-based nanocarriers. Excellent benefits arise from the use of both hydrophobic and hydrophilic chemicals in this arrangement [141,142]. Additionally, by regulating the organic and aqueous phases, the lipid components interact physically to produce a lipid carrier, which then self-assembles into the desired structure [143]. As a result, the manufacture of lipid carriers can be easily scaled up using a variety of viable large-scale production processes [144]. Several soy lecithins made of nanolipids have been created, demonstrating effective drug delivery [145]. Lipid formulations can potentially include essential oils (EEs) [146]. EEs are richer in essential fatty acids, together with monoterpenes and phenylpropenes, and they might be a better alternative carrier than lecithin, given their possible drawbacks. The stability of a lipid carrier prepared using the EE of *Cymbopogon flexuosus* (lemongrass) was improved at room temperature [147].

Moreover, the combination of EEs may increase the quantity of encapsulated hydrophobic medication or vitamins, enabling the sustained release of cargo and improving cell penetration [148]. Compared to standard lipid carriers, lipid nanocarriers produced using linseed oil demonstrated higher drug encapsulation and steady delayed release. Linseed oil was used as a lipid carrier, and thiocolchicoside was found to have better permeability and therapeutic value. Lecithin-Tween 80-glycerol was formulated using palm oil [149], was effective for the delivery of aerosols, and included a significant level of quercetin. The synergy between EE and the loading agent is a crucial consideration in the creation of lipid carriers with EEs. Ginger oil [150], frankincense oil [151], garlic oil [152], and pomegranate seed oil [153] have demonstrated a variety of therapeutic properties, including antibacterial, antioxidant, anticarcinogenic, and calming activities. Thus, EEs are a promising resource for creating useful lipid nanocarriers. Promising nanocarriers have been developed for drug delivery using lipids. Lipid excipients are primarily derived from dietary oils or fats and have a better biodegradation profile [154]. Furthermore, it can pass through the gastrointestinal tract. By improving lymphatic transport, reducing hepatic first-pass metabolism, and inhibiting P-glycoprotein-mediated efflux, lipid-based nanocarriers can raise the bioavailability of a variety of medications [155]. A variety of lipid materials can be classified according to their potential use in pharmaceutical applications. These include natural oils and fats, polyethylene glycol derivatives, cholesterol, phospholipids, fatty acids, fatty acid esters, fatty alcohols, and semisynthetic mono-, di-, and triglycerides (Table 5) [156]. The ability to modify the pharmacokinetic characteristics of lipids allows for the development of various lipid-based nanocarrier types with various applications. However, choosing the best lipid additive for a particular nanocarrier depends on several factors, including purity, chemical stability, physical and chemical properties, solvent capacity, fat digestion, and legal requirements.

### 2.6. Magnetic Nano-Materials in the Treatment of Cancer

**Figure 6:**
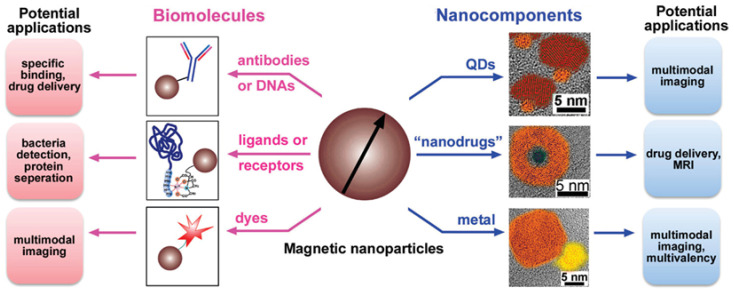
Magnetic nanoparticles with biomolecules and nanocomponents. QDs, quantum dots. This figure is adapted with permission (copyright © 2021, Springer Nature Singapore Pte Ltd., Singapore) from Varghese et al. [184].

Hyperthermic therapy, which involves heating the tumor tissues to a tolerable temperature of 40–43 °C, is currently a successful treatment for tumors. This method effectively destroys tumor cells without having the negative side effects of conventional cancer treatments. On the other hand, conventional treatments can be combined with the hyperthermic therapeutic method. Nearly every branch of research, including hyperthermic therapy, has benefited greatly from the development of nanotechnology. Functionalities made feasible using magnetic nanoparticles (MNPs) are impossible using traditional magnetic materials [183,184]. In addition to having a greater operating temperature range, smaller size, lower toxicity, easier preparation, and lower production costs, newer MNPs also have other benefits. MNPs offer great promise for medical applications owing to a variety of exceptional and distinctive physical and chemical features (Figure 6).

MNPs are employed, in particular, as carriers for targeted drug delivery systems and probes for medical imaging. MNPs are anticipated to play a significant role in the future of cancer diagnostics and precise medication delivery, although further studies are needed to reduce their toxicity and boost their effectiveness [185]. The formation of nanomaterials that can fulfill new biomedical requirements is critical. Highly attractive minutes and high unambiguous surface region proportions of attractive nanoparticles (MNPs) make them suitable for altered drug organization and hyperthermia treatment of diseases. They can likewise upgrade the awareness of biosensors and symptomatic hardware, become specialists in attractive reverberation imaging (X-rays), and perform different roles. The improvement in the accompanying age of MNPs, fitting for these and other biomedical applications, is a consequence of ongoing advances in nanotechnology [186].

By coordinating nanoparticles in the attractive reverberation imaging innovation, sensor gadgets, early malignant growth recognition, and screening can be achieved. These sensors are designated to identify specific biomarkers and substances that might be associated with the turn of events or movement of malignant growth, both during and following the organization of treatments for this normal infection. Utilizing designated in vivo control with an outer attractive field, some of the extraordinary highlights of attractive nanoparticles are broadly utilized in malignant growth treatment as medication conveyance specialists, especially focusing on ideal sites. In addition, to obtain a powerful treatment, customized antineoplastic medicine treatment might be matched with attractive reverberation imaging. Given their distinct chemical nature, the lack of toxicity, biocompatibility, and especially their inducible magnetic moment, the morphological structures of magnetic materials have attracted enormous interest in various scientific domains [187].

MNPs with a mean diameter of 19 nm and a specific absorption rate of 110 ± 30 W/g_Fe3O4_-coated NPs with good biocompatibility to assure impressive stability in physiological media are utilized for magnetic fluid hyperthermia [188]. The extent to which light diminishes clonogenic endurance differs based on the type of radiation utilized, and this decrease is more grounded by MNP cell uptake and hyperthermia therapy. In examples that were presented to MNP uptake, treated with 0.75 Gy carbon-particle light, and exposed to hyperthermia, there were calculable expansions in DNA two-fold strand breaks at 6 h. Hadron irradiation and hyperthermia were combined into the proposed experimental technique. In contrast to conventional procedures, cancer therapy with MNPs offers innovative qualities such as nearly no side effects and a high rate of efficacy. The major goal of targeted medication delivery is to lessen the adverse effects that other common chemotherapies will cause in the body by controlling the potency of the drug at a particular site where tumoral tissue is present [189].

According to Cheng et al., the multimodal strategy offers a way to simultaneously induce significant immunogenicity, reduce safety issues, and monitor immune activity [190]. Indocyanine green (ICG) and the immunostimulant R837 hydrochloride (R837) were loaded into a magnetic nanoparticle delivery method for immunotherapy synergism in cancer. This conveyance strategy consolidates polyethylene glycol polyphenols (DPA-Stake) as the covering layer to stack R837 with Fe_3_O_4_ NPs as the center to stack ICG, resulting in R837-stacked polyphenols covering ICG-loaded magnetic nanoparticles (MIRDs). Upon intravenous infusion, MIRDs deliver extensive flow, X-ray direction, and attractive focusing. The body’s safe reaction was essentially upgraded after the growth, with the arrival of R837 from the external DPA-Stake after the MIRDs with the close infrared (NIR) light causing cancer removal and bringing about the arrival of cancer-related antigens.

For monitoring and measuring the distribution of nanoparticles in biological systems, imaging methods such as magnetic resonance imaging (MRI), computed tomography (CT), PET/SPECT, ultrasound (US), and fluorescence/bioluminescence imaging (FLUO/BLI) are crucial. These techniques guarantee accurate pathology targeting by enabling the real-time observation of nanoparticle uptake, biodistribution, and treatment response [191]. While MRI gives comprehensive anatomical mapping, PET and SPECT offer great sensitivity for identifying radiolabeled nanoparticles [192]. These methods are enhanced by FLUO/BLI imaging, which permits dynamic, non-invasive cellular monitoring [193]. These techniques improve translational results from preclinical models to patients and improve the assessment of nanoparticle-based therapies.

## 3. Nano-Omics

**Figure 7:**
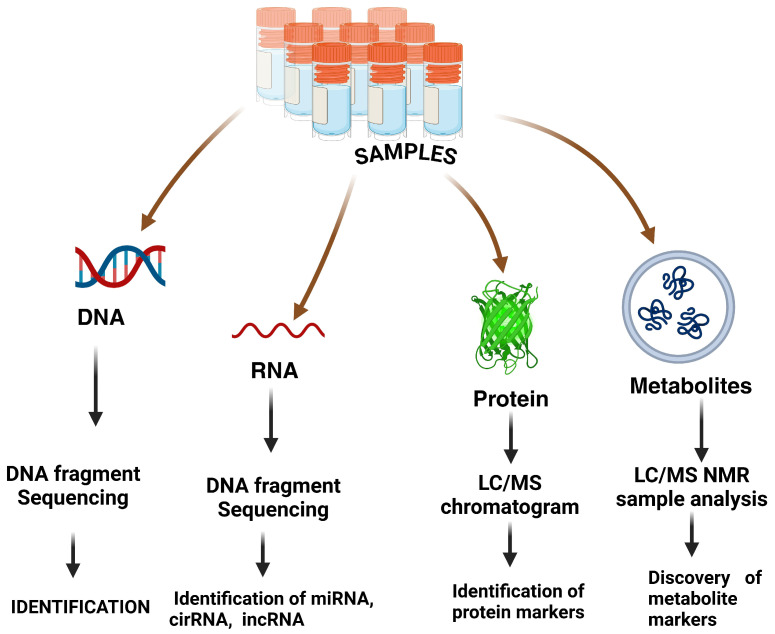
The application of multi-omics technology to identify potential bacterial markers in colorectal cancer (CRC) through DNA sequencing via a metagenomics approach. This figure is reproduced from Dalal et al. [195] and is an open access article (copyright © 2020 by the authors) distributed under the terms and conditions of the Creative Commons Attribution (CC BY)-No Commercial-No Derivatives license.

One of the main goals of cancer studies during the last ten years has entailed the design of “simple” blood tests that enable cancer screening, diagnosis, or tracking, and make it easier to construct individualized therapeutics by avoiding aggressive tumor tissue collection. Results from contemporary biomarker research projects show that, to improve the accuracy and specificity of tests for early-stage cancer detection, numerous markers employed singly or as a component of a multifunctional panel are necessary. The identification of multidimensional (genome, transcriptome, proteome, and metabolome) cancer-associated molecular abnormalities expressed in the blood can identify novel biomarkers and further clarify the underlying molecular processes [194]. By utilizing reinforcement learning, this study developed new hybrid cancer detection methods that include multi-omics workload learning with a SARSA on-policy. It includes collecting clinical data at several levels, such as in-instrument procedures (biopsy, colonoscopy, and mammography) and laboratories at the network’s scattered omics-based clinics (Figure 7).

The study used multi-omics dispersed clinics to analyze the many cancer classifications, including carcinomas, sarcomas, leukemia, and lymphomas, along with their subtypes [195]. A new paradigm for identifying 12,000 gene products (proteins) and over 30,000 phosphosites with a false discovery rate (FDR) of around 1% was applied to the biology of HGG cancer. They assessed two oncogenic receptor tyrosine kinase-driven HGG mice variants by combining integrated planning genetic investigations of the transcriptome and proteome. Abnormalities and/or inactive components in the chimeric genes of neurotrophic tyrosine receptor kinase (NTRK) and platelet-derived growth factor receptor alpha (PDGFRA) are commonly observed in infants and people with HGG [196].

## 4. Environmental Factors and Outcomes for Patients in Rural Areas with the Progression of New Anti-Tumor Interventions

There is increasing agreement that regional variations in cancer incidence are mostly caused by environmental factors. Cancer risk is influenced by a variety of regionally specific factors, including nutrition, lifestyle choices, occupational exposures, and pollution. For example, metropolitan locations have greater rates of lung cancer due to air pollution and exposure to industrial carcinogens, whereas certain regions may have higher rates of gastrointestinal cancer due to dietary variables and infections [197]. Due to less specialist treatment facilities, delayed diagnosis, and restricted access to healthcare services, patients in rural locations frequently face discrepancies in cancer outcomes. Targeted treatments and nanomedicine developments, however, have the potential to close this gap. Drug delivery methods based on nanoparticles might lessen adverse effects and increase the effectiveness of chemotherapy, possibly making it simpler to administer outside of large cancer clinics. Furthermore, early identification and monitoring in rural areas may be made possible by portable diagnostic instruments that use nanotechnology [198]. Geographical constraints may be addressed by combining nanomedicine-based therapies with telemedicine and mobile health units to provide equal access, improving cancer care in underprivileged areas [199]. Supporting the use of these technologies also requires financing for rural healthcare facilities and policy efforts.

Understanding how nanoparticles interact with biological systems—including how they circulate, aggregate, and release medications at tumor sites—requires pharmacokinetic investigations. To ensure optimum tumor targeting, it is helpful to measure characteristics such as circulation half-life, biodistribution, and clearance in order to differentiate between medicines that are free and those that are attached to nanoparticles [200]. By reducing systemic toxicity and increasing therapeutic efficacy, these findings shed light on the regulated release of anti-tumor drugs [201]. It is difficult to assess the full potential of nanomedicine in vivo without comprehensive pharmacokinetic data, which emphasizes the necessity of a thorough analysis throughout preclinical and clinical studies [202].

## 5. Future Perspective

Several cancer detection and treatment studies have made substantial use of nanotechnology, according to different study field groups, and this could be the next great breakthrough in cancer treatment. Although many studies have been conducted in the vicinity, there is still a significant amount of research in cancer nanotechnology that has not come to light. Notwithstanding these limitations, DNA and peptide-based nanomaterials have shown promise for use in cancer therapy. Future developments in this field will significantly improve cancer diagnostics and treatment methods. In addition, it is important to emphasize the functionalization of a multimodal nanostructure that might deliver a double blow to malignancy through rapid detection and efficient treatment. As such, it is evident that nano-oncology will undoubtedly offer a reliable, effective, and secure cancer detection and theragnostic approach in the not-too-distant future.

Nanotechnology has several benefits for medicine, including better imaging for diagnosis and therapy monitoring, less side effects, and accurate medication administration. By overcoming biological barriers, nanoparticles can improve medication accumulation at tumor locations and improve the effectiveness of cancer treatment [203]. The nanoassembly offers several advantages in drug delivery, including carrier-free fabrication with high reproducibility and efficient drug co-loading for adjustable doses. It enables the synchronous co-delivery of DiR and GA with prolonged systemic circulation and self-tracing tumor accumulation. Additionally, it demonstrates effective photothermal conversion. In a 4T1 tumor BALB/c mice xenograft model, Hsp90 inhibition enhances photothermal therapy (PTT). These properties highlight the nanoassembly’s potential for targeted cancer therapy [204].

## 6. Conclusions

Nearly every subject, including physics, biology, chemistry, medicine, and engineering, has benefited from the development of nanotechnology. The medical technology sector is among the most promising in nanotechnology. While chemotherapy and radiation are well-known cancer therapies, they have many limitations, including inaccurate medication targeting and delayed disease identification. Since the drug concentration does not only concentrate on the tumor site but also spreads throughout the body, these approaches frequently fail to treat and track the effects of treatments. To a certain extent, these restrictions can be addressed using nanotechnology. A significant issue in cancer treatment is the targeted tumor site’s precise administration of anticancer medicines, which is mostly achieved by producing such nanopolymers’ as medication delivery systems, such as dendrimers, micelles, and nanocantilevers. By modifying the nanopolymers’ design and structure, we can significantly improve their characteristics and create new, more efficient compounds that are compatible with biological systems. Overall, nano-oncology has created countless opportunities for designing and searching for drugs and drug delivery systems.

## Figures and Tables

**Table 2:** The evidence of various dendrimers used for cancer treatment.

Dendrimers	Conjugated Molecules	Drug	Type of Cancer	Conditions	Ref.
Plain dendrimers					
Poly (propylene imine) dendrimer (PPI)	Chemical conjugation	Melphalan	Breast	In vitro	[47,48]
Polyether dendrimers	D-glucose amine	Methotrexate (MTX)	Gliomas (malignant cancer)	In vitro	[49]
Poly-l-lysine dendrimers (PLL)	-	DOX	Lung and ovarian	In vitro and in vivo	[50]
Melamine dendrimer	Organoiron	Piperazine moieties	Breast	In vitro	[51]
Triazine dendrimer	-	Paclitaxel	Prostate	In vivo and in vitro	[52]
Poly (amidoamine) PAMAM dendrimer	Enzyme linked aptamer	Au-nanoparticles and prostate-specific antigen (PSA)	Prostate	In vitro	[53]
Polyester dendrimer	Manganese	Hypericin	Breast	In vitro	[54]
Citric acid dendrimer	Polyethylene glycol (PEG)	Alendronate	Bone	In vitro	[55]
Dendrimers based on macromolecular monomers					
Peptide dendrimer	Tetra-peptide sequence Gly-Phe-Leu-Gly (GFLG)	DOX	Ovarian	In vivo and in vitro	[53]
Carbohydrate dendrimer	D glucosamine	Methotrexate	Glioma	In vitro	[56]
PEG dendrimers	Di sulfide linkage	PTX and TR3 siRN	Pancreatic	In vitro and in vivo	[57]
Surface engineered dendrimers					
Acetylated dendrimer	Fluorescein isothiocyanate (FITC)	Biotin	Breast	In vitro	[58]
Antibody conjugated dendrimer	J591 anti-PSMA (prostate-specific membrane antigen), fluorophores	-	Prostate	In vitro	[59]
Folate conjugated dendrimer	PAMAM	MTX	Cervical and ovarian	In vitro	[60]
SiRNA conjugated dendrimer	PAMAM	-	Breast	In vivo	[61]
Dendrimers with special property					
Amphiphilic dendrimer	Peptide	DOX	Breast	In vivo and in vitro	[62]
Liquid crystalline dendrimer	Lyotropic	DOX	Ovarian	In vivo and in vitro	[63]
Hybrid dendrimer	Lipid	Paclitaxel	Ovarian	In vivo and in vitro	[64]
Micellar dendrimer	Micelles	Paclitaxel	Breast	In vivo	[65]
Multilingual dendrimer	Functional group	Methotrexate (MTX)	Cervical	In vivo	[66]

**Table 3:** Graphene based nanomaterials.

Graphene-Based Nanomaterials	Properties	Size (nm)	Type of Cancer	Type of Applications	Ref.
Bare graphene-based nanomaterial					
Graphene quantum dots (GQDs)	PTA	41	Cervical, (HeLa), breast	Drug delivery	[82]
Graphene oxide (GO)	Sharp edges/ROS	10–120	Liver	Drug delivery and imaging	[83]
Reduced graphene oxide (rGO)	Sharp edges/ROS	42	Liver	Drug delivery	[84]
Modified GBNMs					
GO-PEG	PTA	10–120	Osteosarcoma	Phytothermal therapy (PTT)	[85]
rGO-HA	PTA	220–240	Liver	PTT	[86]
GO-PEG-FA/GNPs-DOX	PTA/drug carrier	10–120	Human breast	PTT/chemotherapy	[87]
Silver nanostructure-reduced graphene oxide composites (rGO-Ag)	PTA/drug carrier	40–50	Lung	PTT/drug delivery	[88]
PVP-rGO/Bi_2_S_3_@DOX	PTA/RSs carrier	10–120	Cervical	PTT/radiotherapy	[89]
PAH/FA/PEG/GO/siRNA	PTA/gene carrier	10–120	Pancreatic	PTT/gene therapy	[90]
GO-(HPPH)-PEG-HK	PS/carrier	10–120	Synergized immunotherapy	PDT/immunotherapy	[91]
rGO/MTX/SB	PTA/drug carrier	40–50	Metastatic	PTT/chemotherapy/immunotherapy	[92]
GP/AgNW/doped/GP/IrOx	Transparent bioelectronics	-	Colon	PTT/PDT/detecting/chemotherapy	[93]
N-GQD/HMSN/C_3_N_4_/PEG-RGD	PTA	-	-	PTT/PDT/imaging	[94]
GO-Ag nanocomposites	Sharp edges/ROS	~1.2	Human breast	Membrane disruption/oxidative stress/Ag^2+^	[95]
GO-ZnO nanocomposites	Sharp edges/ROS	-	All type of cancer	Membrane disruption/oxidative stress	[96]
DOPA-rGO	Chitosan carrier	750–1000	Breast	Chemo-photothermal therapy	[97]
PAH/FA/PEG/GO/siRNA	PTA/gene carrier	10–100	Pancreatic	PTT/gene therapy	[98]

**Table 4:** CPP-conjugated nanoparticles for improved delivery of anticancer drugs.

CPP-Conjugated/Modified Nanoparticles	Drug	Type of Cancer	Condition	Ref.
CPP-modified lipid nano-capsule	Paclitaxel	GL261, glioma brain tumor	In vivo	[105]
Lysine-rich CPP conjugated with AuNPs	Doxorubicin	Anticancer activity	In vivo	[106]
Mesoporous silica nanoparticles conjugated with CPP	Methotrexate	Brain	In vitro	[107]
PEGlyated liposomes with CPP	Doxorubicin	Human breast adenocarcinoma cells and human fibrosarcoma cells	In vitro	[108]
CPP-modified graphene oxide nanoparticles	Small interfering SiRNA	Breast cancer	In vivo	[109]
Dendrimers conjugated with CPP	Camptothecin	Leukemia	In vivo	[110]
Silver nanoparticles conjugated with CPP	-	Breast adenocarcinoma	In vivo	[111]
PEG-PLA-PMs-HE-CPP	Paclitaxel	Breast cancer	In vivo	[112]
BSA nanoparticle conjugated with CPP(KALA)	Doxorubicin (DOX) Indocyanine green (ICG)	Inhibits tumor activity	Both in vivo and in vitro	[113]
Graphene oxide conjugated with CPP	Doxorubicin	Breast cancer	In vitro	[114]
Arginine-rich CPPs	Doxorubicin loaded liposomes	Ovarian cancer	In vitro	[115]
Solid lipid nanoparticle conjugated with CPP	Docetaxel	Glioblastoma multiforme	In vivo	[116]
PLGA-PEG with CPP	Paclitaxel	Hepatic cancer	In vivo	[117]
Solid lipid nanoparticles surface modified with cyclic peptides	Paclitaxel and naringenin	Glioblastoma multiforme	Both in vivo and in vitro	[118]
Polymersomes conjugated with selective CPP	MTX	Lung cancer	In vivo	[119]

**Table 5:** Lipid-based nanoparticles in cancer therapy.

Conjugated Nano Lipids	Drug	Type of Cancer	Condition	Ref.
Liposomes				
PEGylated liposomes	Doxorubicin	Breast	In vitro	[157]
Charge-reversal cell-penetrating peptide-modified liposomes	Paclitaxel	Melanoma	In vitro	[158]
pH-responsive liposomes	Mitoxantrone	Breast and renal	In vitro	[159]
Estrogen receptor-anchored pH-sensitive liposomes	Doxorubicin	Breast	In vitro	[160]
Anthracycline-conjugated liposomes	Daunorubicin, Doxorubicin, Epirubicin	Breast	In vitro	[161]
Herceptin liposomes	Doxorubicin	Breast	In vivo and in vitro	[162]
Chitosan oligosaccharide-modified liposomes	Paclitaxel	Lung	In vivo and in vitro	[163]
Osimertinib-encapsulated liposomes	Osimertinib	Non small-cell lung	In vitro	[164]
PEGlyated cationic liposomes	Kinesin spindle protein (KSP) siRNA/paclitaxel	Ovarian	In vivo and in vitro	[165]
RGD-modified liposomes	Gemcitabine (GEM)	Ovarian	In vitro	[166]
Aptamer-conjugated liposomes	miRNA-29b	Ovarian	In vitro	[167]
Nano lipid carrier				
Topical nanostructured lipid carrier	Quercetin and resveratrol	Skin	In vivo and in vitro	[168]
Nanostructured lipid carrier	Raloxifene	Breast	In vitro	[169]
Cetuximab functionalized nanostructure lipid carrier	Paclitaxel and 5-Demethylnobiletin (DMN)	Lung	In vivo	[170]
Nanostructured lipid carrier	Curcumin	Breast	In vitro	[171]
Citral-loaded nanostructured lipid	-	Breast	In vivo	[172]
Chitosan-coated nanostructure lipid carrier	Tetrahydro curcumin	Breast	In vitro	[173]
Hyaluronic acid-modified nanostructured lipid carrier	Kaempferol	Non small-lung	In vitro	[174]
Nanostructured lipidic carriers (NLCs)	Ribociclib (RBO)	Breast	In vitro and in vivo	[175]
Calycosin-loaded NLCs	Calycosin	Breast	In vitro and in vivo	[176]
Gefitinib-loaded NLCs	Gefitinib (GEF)	Metastatic lung	In vivo	[177]
Nano-emulsions				
Novel nano-emulsion	5-fluorouracil and Curcumin	Lung	In vivo	[178]
LAP-loaded nano-emulsion (NE-LAP)	Lapachol (LAP)	Breast	In vitro	[179]
Naringenin nano-emulsions	Naringenin (NAR)	Lung	In vivo	[180]
Borage oil based nano-emulsion (B-NE)	Docetaxel (DTX) and thymoquinone (TQ)	Breast	In vitro	[181]
Nano-emulsion-assisted siRNA	-	Pancreatic	In vivo and in vitro	[182]

## Data Availability

Not applicable.
